# Meta-analysis of the association between PADI4 -92C/G polymorphism and rheumatoid arthritis in the Chinese population

**DOI:** 10.1590/1414-431X20176115

**Published:** 2017-08-17

**Authors:** Z.Y. Guo, J.X. Zhang, M. Wu, Y.F. Mei, X.J. Lin, C. Bu, Y. Xie, J. Wang

**Affiliations:** 1 Department of Orthopedic Surgery, The First Affiliated Hospital of Zhejiang University, Hangzhou, China; 2 Department of Orthopedic Surgery, Sanmen People Hospital, Sanmen, China; 3 Department of Orthopedic Surgery, Qingtian People Hospital (The Affiliated Hospital of Lishui Academy), Qingtian, China; 4 Department of Combine of Western Medicine and Traditional Chinese Medicine, Sanmen People Hospital, Sanmen, China; 5 Institute of Virology and Biotechnology, Zhejiang Academy of Agricultural Sciences, Hangzhou, China

**Keywords:** Meta-analysis, Peptidylarginine deiminase 4, Polymorphism, Rheumatoid arthritis

## Abstract

Many studies have evaluated the correlation between peptidylarginine deiminase 4 (PADI4) -92C/G polymorphism and rheumatoid arthritis (RA), but the results remain inconclusive. Therefore, we performed a meta-analysis in the Chinese population to provide comprehensive data on the association between PADI4 -92C/G polymorphism and RA. Eligible studies published before May 2016 were identified in PubMed and Chinese databases. The strengths of these associations were assessed by pooled odds ratios (OR) and 95% confidence interval (CI). Eight studies documenting a total of 1351 RA cases and 1585 controls were included in this meta-analysis. In the overall analysis, a significant association between the PADI4 -92C/G polymorphism and RA was found in the Chinese population (G *vs* C: OR=1.32, 95%CI=1.02–1.71; GG+CG *vs* CC: OR=1.75, 95%CI=1.20–2.53). The subgroup analyses stratified by geographic area(s) and source of controls revealed significant results in South China, in hospital-based studies and population-based studies. In summary, this meta-analysis suggested that PADI4 -92C/G polymorphism may be associated with the RA incidence in the Chinese population, especially for South China. Further studies conducted on other ethnic groups are required for definite conclusions.

## Introduction

Rheumatoid arthritis (RA) is an autoimmune disease characterized by chronic inflammation of the joints, which may lead to joint destruction and disability (1). It is a common chronic inflammatory rheumatic disease, with a prevalence estimate of 1% in the world (2,3). Although the etiology and pathogenesis of RA are not clearly defined, a genetic component of RA susceptibility has been established by twin and family studies, which estimated that the heritability of RA liability might be as high as 60% (4). Therefore, genetic factors such as single nucleotide polymorphisms might play important roles in RA pathogenesis (5). Many potential RA susceptibility genes have been identified in recent years. Peptidylarginine deiminase 4 (PADI4) is one of these genes, which is mainly distributed in the cells of various hematopoietic lineages, and expressed at high levels in the inflamed synovium of patients with RA. The PADI4 gene is located on chromosome 1p36 and several polymorphisms have been identified in its promoter. PADI4 -92C/G (or rs874881) single nucleotide polymorphism has been one of the most extensively examined in studies on PADI4 polymorphisms in RA. Some studies have attempted to clarify this relationship between PADI4 -92C/G polymorphism and RA risk, but there has been no definite consensus to date. Differences in study results may be due to ethnic and geographical heterogeneity of the patients studied, as well as the limited number of patients included in each study. To better address the association between PADI4 -92C/G polymorphism and RA risk, we performed a meta-analysis of all eligible studies conducted in the Chinese population.

## Material and Methods

### Search strategy and selection criteria

Eligible studies were identified by searching PubMed and Chinese databases for relevant reports published before May 2016 using the following search terms: PADI4, peptidylarginine deiminase 4, -92C/G, rheumatoid arthritis, Chinese, China and Taiwan. No restriction was imposed on language. Furthermore, references cited in the retrieved articles were screened to trace additional relevant studies.

Inclusion criteria were: 1) case-control or cohort studies describing the association of the PADI4 -92C/G polymorphism and RA; 2) provided the genotypes in cases and controls; 3) all patients diagnosed according to the classification criteria proposed by the American College of Rheumatology for RA in 1987; 4) participants were Chinese. Exclusion criteria were: 1) repeated literature; 2) incomplete data; 3) case-only articles; 4) review articles and abstracts.

### Data extraction

We conducted a systematic review and meta-analysis in accordance with the guidelines provided by the Preferred Reporting Items for Systematic Reviews and Meta-Analyses (PRISMA) statement. All studies were reviewed twice and the data was extracted using a standardized form. Disagreements were resolved by discussion. All the information was collected from each included article: name of the first author, publication year, source of controls, geographic area(s), sample size, and PADI4 -92C/G genotypes data. Hardy-Weinberg equilibrium (HWE) in controls was calculated from corresponding genotype distributions. In this meta-analysis, the quality of individual studies was assessed according to the nine-star Newcastle-Ottawa Scale (http://www.ohri.ca/programs/clinical_epidemiology/oxford.asp).

### Statistical analysis

The strength of the association between PADI4 -92C/G polymorphism and RA susceptibility was estimated by calculating the pooled odd ratios (OR) with their 95% confidence intervals (CI). The Z-test was used to determine the significance of the pooled OR and 95%CI. The between-study heterogeneity was assessed using the chi-square based Q-statistic (6). The random-effects or fixed-effects model was used according to the studies heterogeneity. The fixed-effects method assumes no significant heterogeneity between the results of the individual studies being pooled, whereas the random-effects method allows for such heterogeneity. The fixed-effects and random-effects methods were used according to Mantel-Haenszel (7) and DerSimonian and Laird methods (6), respectively. We compared the results of fixed-effects model and random-effects model to evaluate the sensitivity of our analysis (8). For exploring sources of heterogeneity, stratified analyses according to geographic areas and source of controls were also performed. All statistical analyses were conducted using the Stata 10.0 software (StataCorp, USA).

## Results

### Eligible studies

**Figure 1.**
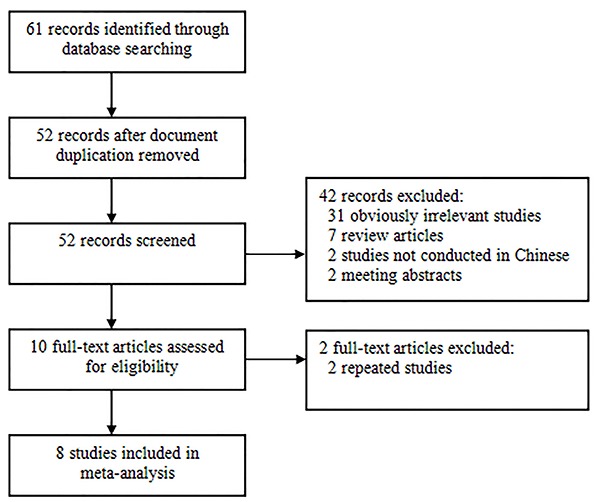
Flow diagram of the literature search.

A total of 52 articles that examined the association between PADI4 polymorphisms and risk of RA were identified after duplicates were removed in different databases. After the first screening of titles and abstracts, 42 articles were excluded due to the exclusion criteria. Of the 10 potentially relevant articles (9–18) identified for full study retrieval, two (9,10) were excluded due to repeated studies. Finally, 8 case-control studies (11–18) met the inclusion criteria (Figure 1). The publication year of involved studies ranged from 2007 to 2012. A total of 1351 RA cases and 1585 controls were included in this meta-analysis. Characteristics of included studies are summarized in Table 1.

**Table 1.** Characteristics of studies included in the meta-analysis.

Reference	Source of controls	Geographic location	Case (n)	Control (n)	Cases			Controls			HWE		Quality score
					CC	CG	GG	CC	CG	GG	χ^2^	P	
Lu 11 (2007)	PB	Shanghai	41	56	5	36	0	42	14	0	1.14	0.285	7
Fan 12 (2008)	PB	Shanghai	70	81	28	31	11	41	25	15	7.88	0.005	7
Wen 13 (2009)	PB	Hebei	105	96	24	60	21	40	44	12	0.00	0.985	8
Zhong 14 (2010)	PB	Chongqing	302	322	89	143	70	107	157	58	0.00	0.975	8
Chen 15 (2011)	PB	Shanghai	378	204	131	167	80	95	61	48	27.69	0.000	7
Cheng 16 (2012)	HB	Jiangsu	312	694	96	145	71	243	338	113	0.06	0.803	8
Liu 17 (2012)	PB	Qinghai	90	90	27	49	14	26	27	37	13.75	0.000	7
Li 18 (2012)	HB	Inner Mongolia	53	42	20	27	6	22	16	4	0.19	0.666	8

PB: population-based; HB: hospital-based; HWE: Hardy-Weinberg equilibrium.

### Meta-analysis

**Figure 2.**
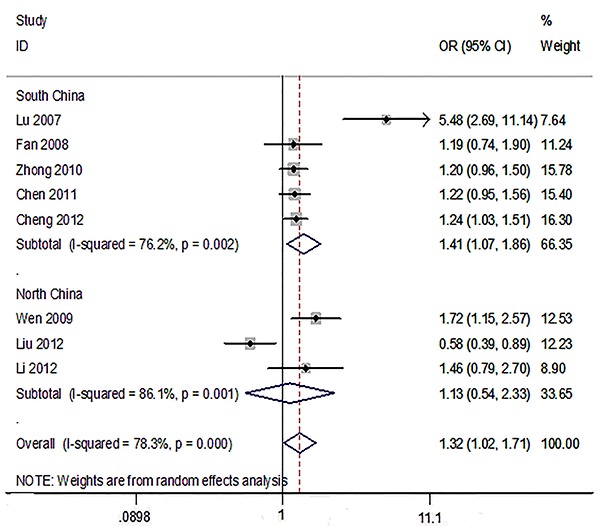
Forest plot of all selected studies on the association between PADI4 -92C/G polymorphism and rheumatoid arthritis risk in Chinese (for allele model G *vs* C). See Table 1 for corresponding reference numbers.

In the total analyses, the combined results revealed that the G variant of the PADI4 -92C/G gene polymorphism was significantly associated with an increased risk of RA in the Chinese population (for G *vs* C: OR=1.32, 95%CI=1.02–1.71; for GG+CG *vs* CC: OR=1.75, 95%CI=1.20–2.53; Figure 2; Table 2). Furthermore, we performed the analysis by excluding the HWE-violating studies. The results suggested that the G variant of the GG genotype was significantly associated with RA in all models (for G *vs* C: OR=1.62, 95%CI=1.16–2.26; for GG *vs* CC: OR=1.63, 95%CI=1.25–2.14; for GG *vs* CC+CG: OR=1.47, 95%CI=1.16–1.86; for GG+CG *vs* CC: OR=2.22, 95%CI=1.21–4.10; Table 2).

**Table 2.** Association of the PADI4 -92C/G gene polymorphism and rheumatoid arthritis susceptibility.

Analysis model		n	ORr (95%CI)	ORf (95%CI)	P_h_
G *vs* C	Total analysis	8	1.32 (1.02–1.71)	1.24 (1.12–1.39)	0.000
	In HWE	5	1.62 (1.16–2.26)	1.35 (1.19–1.54)	0.001
	Population-based	6	1.36 (0.94–1.97)	1.23 (1.08–1.41)	0.000
	Hospital-based	2	1.26 (1.05–1.51)	1.26 (1.05–1.51)	0.634
	South China	5	1.41 (1.07–1.86)	1.28 (1.14–1.44)	0.002
	North China	3	1.13 (0.54–2.33)	1.09 (0.84–1.41)	0.001
GG *vs* CC	Total analysis	7	1.28 (0.88–1.85)	1.33 (1.07–1.65)	0.027
	In HWE	4	1.63 (1.25–2.14)	1.63 (1.25–2.14)	0.575
	Population-based	5	1.16 (0.69–1.96)	1.21 (0.93–1.58)	0.012
	Hospital-based	2	1.59 (1.11–2.30)	1.59 (1.11–2.30)	0.960
	South China	4	1.40 (1.11–1.77)	1.40 (1.11–1.77)	0.753
	North China	3	1.17 (0.28–4.84)	1.04 (0.62–1.75)	0.002
GG *vs* CC+CG	Total analysis	7	0.99 (0.65–1.52)	1.10 (0.91–1.13)	0.001
	In HWE	4	1.47 (1.16–1.86)	1.47 (1.16–1.86)	0.935
	Population-based	5	0.87 (0.50–1.52)	0.95 (0.75–1.20)	0.001
	Hospital-based	2	1.50 (1.08–2.06)	1.49 (1.08–2.06)	0.751
	South China	4	1.19 (0.88–1.60)	1.23 (0.99–1.51)	0.144
	North China	3	0.80 (0.21–3.03)	0.68 (0.43–1.06)	0.001
GG+CG *vs* CC	Total analysis	8	1.75 (1.20–2.53)	1.49 (1.27–1.74)	0.000
	In HWE	5	2.22 (1.21–4.10)	1.50 (1.24–1.82)	0.000
	Population-based	6	1.97 (1.17–3.30)	1.62 (1.33–1.97)	0.000
	Hospital-based	2	1.27 (0.97–1.66)	1.27 (0.97–1.66)	0.363
	South China	5	1.87 (1.14–3.09)	1.47 (1.23–1.74)	0.000
	North China	3	1.60 (0.89–2.88)	1.60 (0.93–2.36)	0.112

ORr: Odd ratio for random-effect model; ORf: Odd ratio for fixed-effect model; P_h_: P value for heterogeneity test; HWE: Hardy-Weinberg equilibrium. North China included Hebei, Qinghai and Inner Mongolia. South China included Shanghai, Chongqing and Jiangsu.

In the subgroup analysis stratified by source of controls, there were significantly increased risks both in the population-based analysis (GG+CG *vs* CC: OR=1.97, 95%CI=1.17–3.30) and the hospital-based analysis (G *vs* C: OR=1.26, 95%CI=1.05–1.51; GG *vs* CC: OR=1.59, 95%CI=1.11–2.30; GG *vs* CC+CG: OR=1.49, 95%CI=1.08–2.06). In the subgroup analysis stratified by geographical areas, there were significantly increased risks in South China (G *vs* C: OR=1.41, 95%CI=1.07–1.86; GG *vs* CC: OR=1.40, 95%CI=1.11–1.77; GG+CG *vs* CC: OR=1.87, 95%CI=1.14–3.09), but not in North China (Table 2; Figure 2).

### Sensitivity analysis

To evaluate stability, we compared the pooled results from fixed-effects model and random-effects model. The significant pooled ORs were not altered between the two models (Table 2). Therefore, the sensitivity analysis suggested that the data in this meta-analysis are relatively reliable.

## Discussion

Convincing evidence has emerged indicating that individual susceptibility to RA is partially determined by a number of genetic variations. The relationship between PADI4 -92C/G polymorphisms and RA risk attracted the attention of both doctors and researchers. Since the first positive association between PADI4 -92C/G and RA was reported in a Japanese population (19), a number of studies have reported the same association, but the results were inconclusive. Recently, several meta-analyses regarding PADI4 -92C/G polymorphism and RA risk have been published ([Bibr B20] 21–22). Of these, two meta-analyses (20,22) found that the PADI4 -92C/G polymorphism had a positive association with RA in Asians, but not in Caucasians, while Yang et al. (21) found a significant result only in Africans. Therefore, we conducted this meta-analysis to derive a more precise estimate of the association between PADI4 -92C/G polymorphism and susceptibility to RA in the Chinese population and lessen the impact of regional and racial differences.

From the combined statistical results, we found a significant association between PADI4 -92C/G polymorphism and RA risk in the total analyses. The controls in three cohorts (12,15,17) were not consistent with HWE (P<0.05), which contributed to more than 20% of the controls included in the study. Therefore, we further performed the analysis by excluding HWE-violating studies in control groups, and the results of a significant association were maintained.

When stratifying by source of controls, significant associations were found in hospital-based studies as well as in population-based studies with only one analysis model (GG+CG vs CC), suggesting that the same gene polymorphism may have different roles in RA susceptibility among different controls. By dividing the samples into subgroups according to geographic areas, a significant association was found in South China, but not in North China, showing that there is a higher susceptibility of PADI4 -92C/G variant in southerners than in northerners of China. This may suggest a possible role of geographic differences in genetic backgrounds and the environment people live in. Because of insufficient data, however, we were unable to perform subgroup analysis for ethnic and other environmental factors. Therefore, studies of RA-related genes should be based on region and nationality, and the control subjects should be comparable with the cases in all relevant aspects.

Heterogeneity is a potential problem in understanding the results of meta-analyses. In this study, significant heterogeneity between different studies was observed in the overall population. To clarify the source of heterogeneity, geographic areas and control source were used to stratify the studies, finding that part of this heterogeneity can be effectively attenuated or removed when stratifying by geographic areas. This indicates that it is important for meta-analyses of genetic association studies to perform subgroup analyses by geographic areas. After subgroup analysis by source of controls, the heterogeneity was also decreased; therefore, it can be assumed that the heterogeneity partly results from differences in source of controls. That may be because potential confounding factors in many epidemiologic studies result from the difference in control types (23). Furthermore, other factors should also be explored to identify the source of heterogeneity if more data are available.

Several limitations of this study should be noted. First, this ethnic-specific meta-analysis only included data from Chinese patients, and thus our results are only applicable to this ethnic group. Second, in the subgroup analyses by geographical areas and source of controls, the sample size was relatively small and the statistical power might be insufficient. Third, due to the limitations of funnel plotting, which requires a range of studies, we did not evaluate publication bias in this meta-analysis.

In conclusion, this meta-analysis suggested that PADI4 -92C/G polymorphism may be associated with the RA incidence in the Chinese population, and may play a stronger role in southerners than in northerners of China. More studies are warranted to assess this association in other ethnic groups.
